# EBV-miR-BART7-3p Imposes Stemness in Nasopharyngeal Carcinoma Cells by Suppressing SMAD7

**DOI:** 10.3389/fgene.2019.00939

**Published:** 2019-10-17

**Authors:** Longmei Cai, Yufei Long, Tuotuo Chong, Wenzhi Cai, Chi Man Tsang, Xiaohan Zhou, Yanling Lin, Tengteng Ding, Wenyan Zhou, Hongli Zhao, Yuxiang Chen, Jianguo Wang, Xiaoming Lyu, William C. Cho, Xin Li

**Affiliations:** ^1^Shenzhen Key Laboratory of Viral Oncology, The Clinical Innovation & Research Center (CIRC), Shenzhen Hospital, Southern Medical University, Shenzhen, China; ^2^Department of Radiation Oncology, Nanfang Hospital, Southern Medical University, Guangzhou, China; ^3^Department of Anatomy and Center for Cancer Research, LKS Faculty of Medicine, The University of Hong Kong, Hong Kong; ^4^Department of Clinical Oncology, Queen Elizabeth Hospital, Kowloon, Hong Kong

**Keywords:** EBV-miR-BART7-3p, Epstein-Barr virus, microRNA, nasopharyngeal carcinoma, SMAD7, stemness

## Abstract

Cancer stem-like cells, possessing “stemness” properties, play crucial roles in progression, metastasis, and drug resistance in various cancers. Viral microRNAs (such as EBV-miR-BART7-3p), as exogenous regulators, have been discovered to regulate malignant progression of nasopharyngeal carcinoma (NPC), suggesting a possible role of viral microRNAs in imposing stemness. In this study, we found that EBV-miR-BART7-3p induce stemness of NPC cells. We firstly reported that EBV-miR-BART7-3p increased the percentage of side population cells, the development of tumor spheres, and the expression level of stemness markers *in vitro*. This viral microRNA also enhanced stem-like or cancer-initiating properties of NPC cells *in vivo*. Besides, we identified SMAD7 as a novel target gene of EBV-miR-BART7-3p in addition to PTEN gene we previously reported; this viral microRNA suppressed SMAD7, led to activation of TGF-β signaling, and eventually enhanced the stemness of NPC cells. Silencing of SMAD7 resembled the effects generated by EBV-miR-BART7-3p in NPC cells. After reconstitution of SMAD7, EBV-miR-BART7-3p-expressing cells underwent a phenotypic reversion. EBV-positive NPC cells were used to enable experimental validation. Finally, we further discovered that EBV-miR-BART7-3p increased chemo-resistance of NPC *in vitro* and *in vivo*, supporting that EBV-miR-BART7-3 resulted in increased stemness of NPC cells and lead to drug resistance and cancer recurrence. Overall, this study uncovered a novel mechanism underlying viral microRNA-associated stemness of NPC cells. This viral microRNA and its associated cellular genes may be potential therapeutic targets for restraining chemo-resistance and recurrence of NPC.

## Introduction

Nasopharyngeal carcinoma (NPC) is an Epstein–Barr virus (EBV)-associated malignancy arising from the nasopharynx. There is an unbalanced distribution of its morbidity in different regions of the world with a high incident rate of approximately 30 per 100,000 being observed in Southern China, Southeast Asia and North Africa (Spano et al., 2003; Cho, 2010). Currently, concurrent chemo-radiotherapy (CCRT) are the preferential treatments for NPC. Despite improved diagnostic and therapeutic strategies, 30–60% of NPC progresses to be a recurrent and chemoradiotherapy-resistant disease.

The “stemness” refers to the integrated functioning of molecular programs that govern and maintain the stem cell state. Cancer stem-like cells are a small population of cells within tumor holding “stemness” properties that facilitate tumor growth, invasion, and treatment failure (Aponte and Caicedo, 2017). Deciphering its underlying mechanisms may be helpful in the development of therapeutic intervention of recurrence and drug resistance of NPC.

EBV is one of the major etiological factors for NPC tumorigenesis. It encodes the oncogenic proteins (LMP1, LMP2, EBNA1, BARF1), EBERs, and 25 viral microRNA precursors in latently infected cells. LMP1 can induce cancer stem/progenitor-like cells in nasopharyngeal epithelial cell lines (Kondo et al., 2011). LMP-2A can prompt cell migration, epithelial-to-mesenchymal transition (EMT) and stemness in NPC (Kong et al., 2010). However, the expression levels of these EBV-encoded proteins are usually low and sometimes even undetectable in NPC cases, suggesting other potential regulatory factors might be participating in the recurrence and progression of NPC (Cho, 2007).

EBV-BART-microRNAs, encoded from the BamHI-A rightward transcript (BART) of the EBV genome, are abundantly expressed in all NPC tissues (Marquitz and Raab-Traub, 2012). They play important roles in maintaining persistent and latent EBV infection by suppressing the host immune response and facilitating epithelial transformation by perturbing signal transduction pathways in the pathogenesis of NPC (Verhoeven et al., 2016). Notably, we previously found that EBV-miR-BART7-3p was able to promote the proliferation, metastasis and EMT of NPC cells by targeting an important tumor suppressor PTEN and its associated signals (Cai et al., 2015a). Because tumor cells with stemness are usually associated with high ability in EMT and high resistance to chemotherapy (Shibue and Weinberg, 2017), we hypothesized that EBV-BART-microRNAs may also induce stem-like phenotypes and contribute to failure of treatment in NPC. Therefore, this study aimed to further elucidate the role of EBV-miR-BART7-3p as an oncomir to promote stemness in NPC.

Small mothers against decapentaplegic (SMAD) proteins are intracellular regulatory factors that transduce extracellular signals from TGF-β ligands to the nucleus (Derynck and Zhang, 2003; Macias et al., 2015). They have been subdivided into three groups: (1) receptor-associated SMADs (R-SMADs; SMAD1, 2, 3, 5, 8) are direct substrates of TGF-β family receptor kinases (Ten and Arthur, 2007); (2) common SMAD (co-SMAD; SMAD4) binds to the activated R-SMADs to form a complex that translocates into the nucleus (Massague and Wotton, 2000), and (3) inhibitory SMADs (I-SMADs: SMAD 6, 7) antagonize the signaling of the other groups, thus exerting an inhibitory effect on TGF-β superfamily pathways by various mechanisms (Wakefield and Hill, 2013; Zhong et al., 2013). SMAD2 and SMAD3 are the most important proteins in the SMAD family. Activated TGF-βRI phosphorylates cytoplasmic SMAD2 and SMAD3 (SMAD2/3) transcription factors, allowing them to be translocated into the nucleus and participate in tumor development.

TGF-β and SMAD3 tropic cytokine have the ability to affect a range of biological functions and activities such as proliferation, differentiation, migration, apoptosis, cell adhesion, and regulation of EMT and maintenance of stemness (Xu et al., 1999; Li et al., 2017b). TGF-β, Wnt, PI3K/AKT signaling have been reported contributing to tumor growth. Despite the emerging evidence of TGF-β and the SMADs in regulating epithelial transformation, their roles in promoting stemness of NPC remain unclear.

In this study, we report that EBV-miR-BART7-3p imposes NPC stemness. This viral microRNA increases the percentage of side population cells, the formation of tumor spheres, and the expression level of stemness markers in NPC cells *in vitro*. It also enhances stem-like or cancer-initiating properties of NPC cells *in vivo*. Importantly, this viral microRNA suppresses a novel target SMAD7 in NPC, activating TGF-β signaling and enhancing NPC stemness. Furthermore, this viral microRNA is found to induce chemo-resistance of NPC, supporting that it is associated with NPC stemness, and thereby leads to higher drug resistance and cancer recurrence. Therefore, this study displays a novel mechanism underlying viral microRNA-associated stemness of NPC cells, providing a potential theoretical basis for targeted therapy of NPC.

## Materials and Methods

### Ethics Statement

Patient’s informed written consents and the Ethics Committee approval of Southern Medical University have been received before collecting clinical tissues. The Ethical Committee for Animal Research of the Southern Medical University approved our animal experiments (No. 44007200042655), which were organized by the state guidelines from the Ministry of Science and Technology of China.

### Clinical Tissue Specimens and Cell Lines

Sixty-two tumor specimens were obtained from undifferentiated NPC patients who had not treated with radiotherapy or chemotherapy. Twenty normal nasopharyngeal (NP) specimens from non-NPC individual were collected respectively from the Zhongshan People’s Hospital and the Nanfang Hospital (Guangdong, China) for clinical data analyses (**Supplementary Tables 1** and **2**). Staging was performed according to the 1992 Fuzhou NPC staging system of China. Four NPC cell lines (CNE2, 5-8F, HONE1 and HONE1-EBV) were obtained from the Cancer Research Institute of Southern Medical University. Two NPC cell lines (HK1 and HK1-EBV) were kindly provided by Professor SW Tsao from the University of Hong Kong.

### Tumor Sphere Assay

CNE2 and 5-8F cells were cultured in serum-free tumorspheric medium [DMEM-F12, B27 (1:50), EGF (20 ng/ml), bFGF (20 ng/ml)] on ultra-low attachment plates (Corning, Shanghai, China) at 37 °C in a humidified atmosphere of 95% air and 5% CO_2_. Cultures were fed weekly and passaged every 2 weeks. CNE2 and 5-8F tumor spheroids were observed using a Nikon Eclipse fluorescence microscope (Nikon, Japan).

### SP Analysis and Cell Sorting

Adherent cells (1×10^6^ cells/ml) were resuspended in ice-cold RPMI 1640 culture (supplemented with 2% fetal bovine serum (FBS). Hoechst 33342 (Sigma-Aldrich, St. Louis, MO) was then added at a final concentration of 5 mg/ml and the samples were incubated for 90 min in the dark with periodic mixing. The cells were then washed twice with PBS, and 1 mg/ml propidium iodide (Sigma-Aldrich) was added. Data were acquired and sorted using BD FACS Ariaflow cytometer (BD Biosciences, USA). Data analyses were done using FlowJo software (FlowJo LLC, Ashland, Oregon, USA).

### Extraction of Total RNA and Quantitative RT-PCR

Total RNA extraction, mature miRNAs reverse transcription and quantification of interested genes were performed as our early study (Cai et al., 2015b). The primers used in this study were listed in **Supplementary Table 4**. Quantification of EBV-miR-BART7-3P and SMAD7 were conducted with Takara microRNA/RNA assays (Applied Biosystems, Dalian, China). RNU6B and GAPDH were used for normalizing the expression of miRNA and mRNA, respectively. The fold changes were calculated by using the 2¯^△△Ct^ method.

### Cell Culture and Transfections

The NPC cell lines were cultured in 10% newborn calf serum (NCS; Hyclone, Invitrogen) RPMI-1640 (Invitrogen), and were maintained at 37 °C with 5% CO_2_. For instant transfection, the medium was changed to Opti-Mem (Gibco) with 10% FBS (Gibco). Cells were seeded on six-well plates (JET, China) 24 h before transfection. siRNA-control and siRNA-SMAD7 was transiently transfected into cells using Lipofectamine^™^ 2000 (Invitrogen) in serum-free conditions (**Supplementary Tables 6**). After 5 h, the medium was changed to fresh RPMI-1640 with 10% FBS.

Lentivirus (GV209, H1-MCS-CMV-EGFP) particles carrying EBV-miR-BART7-3p precursor and its control were purchased from GeneChem (Shanghai, China) (**Supplementary Tables 3**). The lentiviral transductions were carried out following the manufacturer’s protocol. The resulting cells were seeded onto 24-well plates and cultured for 3 weeks to obtain stable EBV-miR-BART7-3p overexpressing cells and control cells. The high expression of EBV-miR-BART7-3p was detected by FACS and quantitative RT-PCR.

### Western Blot

Western blot analyses were performed with standard methods. All antibodies used for Western blot were listed in **Supplementary Table 5**. Cell pellets were lysed in RIPA buffer containing protease and phosphatase inhibitors (Sigma-Aldrich). After that they were separated in 10% sodium dodecyl sulfate-polyacrylamide gel electrophoresis (SDS-PAGE). For immunoblotting, the selected gel lanes were cut and placed in wet filter prepared blot modules, then total proteins in gel were transferred to covering PVDF membrane (Millipore, Billerica, MA, USA) incubating in the electrophoresis chamber. The membranes were blocked by 5% BSA for 1 h at room temperature and incubated overnight in suggested primary antibody at 4°C. After washing with TBST, the membranes were incubated with HRP secondary antibodies for 1 h at room temperature except for anti-SOX2 antibody as it has been conjugated with HRP and can be visualized by ECL directly. The bands were visualized and captured by ECL Western blot kit (CWBIO Technology, Beijing, China) and ChemiDoc CRS+ Imager (Bio-Rad, Hercules, CA, USA), respectively.

### Dual Luciferase Assay

The luciferase reporter (the pEZX-MT01 vector, GeneCopoeia) that contained either the wild-type or mutant 3′UTR of Smad7 was produced by GeneCopoeia (Guangdong,China). EBV-miR-BART7-3p mimic or negative control (NC) (Genepharma, Shanghai, China) and luciferase reporter were co-transfected into NPC cells (1×10^4^) 48 h before luciferase activities were analyzed with a Dual-Luciferase Reporter Assay System (Promega, Madison, WI, USA). Cells were also co-transfected with 20 nM anti-miR or anti-C (Genepharma, Shanghai, China) for antagonism experiments (**Supplementary Table 3**).

### Immunohistochemistry

The paraffin sections prepared from *in vivo* experiments were applied to immunohistochemistry assays for detecting protein expression levels of SMAD7 proteins. The indirect streptavidin-peroxidase method was used as the manufacturer’s introduction. Paraffin sections were deparaffinized in xylene and rehydrated sequentially in ethanol. For antigen retrieval, paraffin sections were incubated in sodium citrate buffer at 100°C for 15 min, then quenched in 3% hydrogen peroxide to block endogenous peroxidase activity for 10 min at room temperature and washed in TBST. Slides were blocked in 5% BSA for 1 h at room temperature, then incubated overnight in primary antibody (1:200) at 4°C. After washes with TBST for three times, slides were incubate with secondary antibody in a humidified chamber for 30 min or longer and wash 5 min in buffer as before.

### Confocal Microscope Examinations

NPC cell lines were seeded on sterile glass bottom dishes, washed with PBS after 12 h and fixed with 4% paraformaldehyde for 15 min. Cells were treated with 0.3% Triton X-100 for blocking before incubated with primary monoclonal antibodies (p-SMAD2 and p-SMAD3) in 1% BSA for 2 h at room temperature. Secondary antibodies were incubated in dark after three washes with PBS. DAPI was used for nuclei staining. Images were obtained on the Olympus confocal micrograph system, and analyzed with FV10-ASW1.7 viewer software (Olympus, Tokyo, Japan).

### *In Vitro* Drug Susceptibility Test (MTT Assay)

CNE2-BART7-3P, 5-8F-BART7-3P and the control group cells were seeded on 96-well cell culture plates (NEST) at 1,000 cells per well in 200 ul of growth medium and allowed to adhere overnight. The medium was replaced with fresh one that contained the tested drugs at concentrations of 0, 5, 10, 100, 1,000, 2,000, and 5,000 ng/ml.

After incubation with fresh medium containing sterile MTT dye (5 mg/ml) in standard conditions for 4 h, the MTT solution was aspirated, and 150 μl of dimethyl sulfoxide (DMSO) was added to dissolve the formazan crystals for 20 min. Spectrometric absorbance at 490 nm was measured by BioTek EL×800 microplate photometer (BioTek Instruments, Inc, Winooski, VT, USA).

### *In Vivo* Tumorigenesis in Nude Mice

Four to five week old male mice (18–20 g in weight) from the Central Animal Facility of Southern Medical University were used. A total of 1 × 10^6^ CNE2-BART7-3p and CNE2-NC in 0.2 ml RPMI 1640 medium were subcutaneously injected into the left and right sides of the back of each mouse respectively. The tumor sizes were measured periodically and calculated using the formula = 0.5×a×b^2^ (a and b were the long and short diameters of the tumors respectively). Every mouse was treated by intraperitoneal injection with cisplatin (5 mg/kg, every 3 days) when the long diameter of the tumor reached 10 mm. Mice were sacrificed after 3 weeks post treatment. Tumors were fixed with 4% paraformaldehyde solution and stored at 4°C.

### Statistical Analysis

Each data was showed from at least three independent experiments using the mean ± s.e.m.

Comparisons between two groups were performed using Student’s *t*-test, unless otherwise indicated. The association between EBV-miR-BART7-3p and SMAD7 gene was analyzed using Spearman’s correlation coefficient. Statistical analyses were performed with the SPSS 13.0 statistical software package (SPSS Inc. Chicago, IL, USA). All statistical tests were two-sided, and P < 0.05 was considered to be statistically significant.

## Results

### EBV-miR-BART7-3p Induces Stem-Like Properties in EBV-Positive NPC Cells

**Figure 1:**
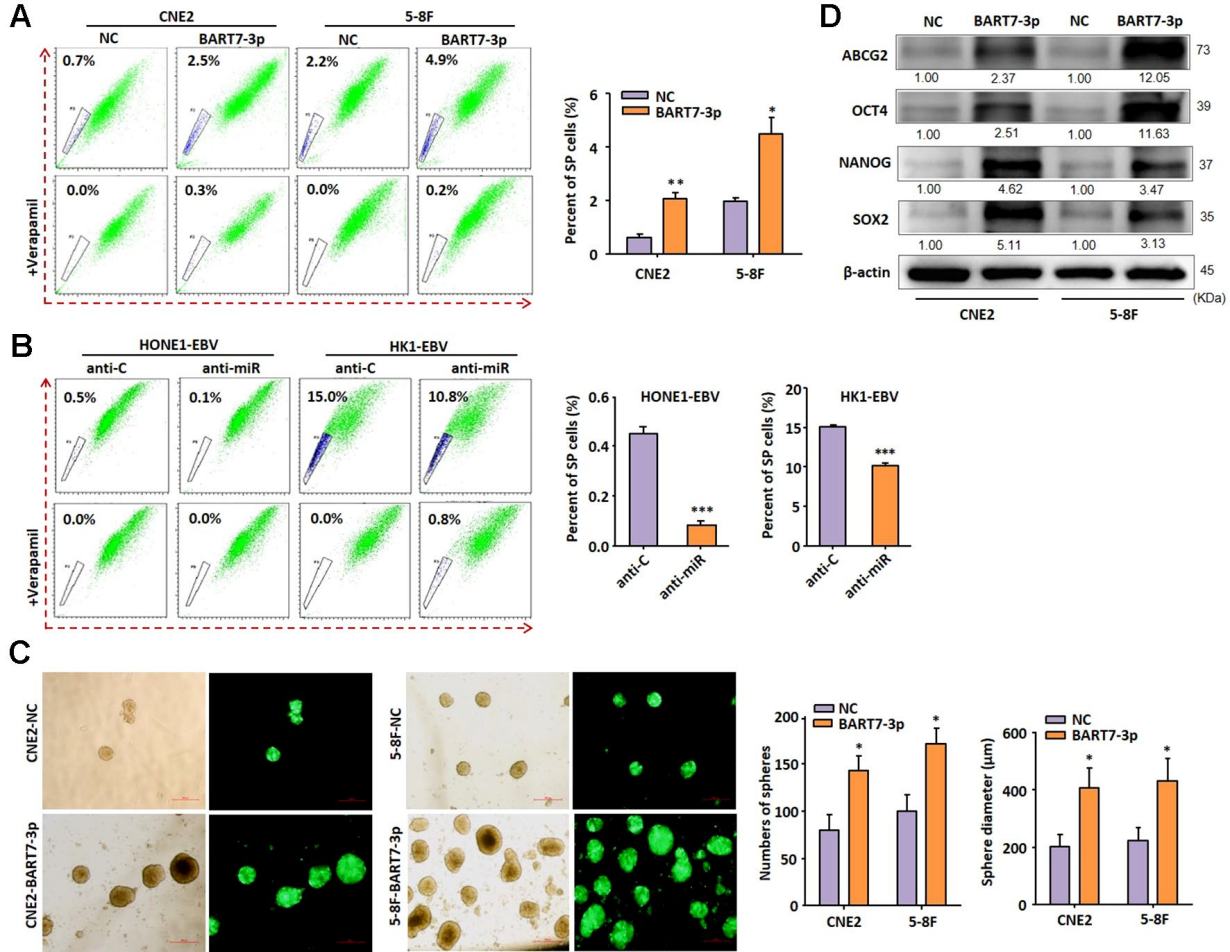
EBV-miR-BART7-3p induces stem-like properties in EBV-positive NPC cells. **(A)** Flow cytometric profiles of SP cells among the CNE2 and 5-8F NPC cell lines after stable expression of BART7-3p and incubated with Hoechst 33342. SP cell profiles in the presence of verapamil are shown in the bottom panels. The percentages of SP cells are indicated. **(B)** Flow cytometric profiles of SP cells within the EBV+HONE1and EBV+HK1 cell populations after treatment of anti-C or anti-miR and incubated with Hoechst 33342. The percentages of SP cells are indicated. **(C)** Images showing tumor sphere formation in CNE2 and 5-8F NPC cells expressing EBV-miR-BART7-3p (Magnification, ×20; scale bar, 500 um). Columns in chart show number of spheres counted in 9 fields for each group; 0, P < v0.05. **(D)** Western blot analysis shows up-regulated expression of the stem cell markers ABCG2, OCT4, Nanog, SOX2 at protein level (*P < 0.05, **P < 0.01, ***P < 0.001).

To examine the functional properties of EBV-miR-BART7-3p, two EBV-negative NPC cell lines (CNE2 and 5-8F) stably expressing EBV-miR-BART7-3p were generated by lentivirus-mediated transduction (**Supplementary Figure 1A**). The expression levels of EBV-miR-BART7-3p in CNE2 and 5-8F were comparable to that in pooled NPC tissue derived from clinical patients (**Supplementary Figure 1B**). It has been reported that EMT is a common feature of cancer stem cells or cancer initiating cells (Puisieux et al., 2014). Moreover, our previous study has shown that EBV-miR-BART7-3p was able to induce EMT in NPC cells (Cai et al., 2015b). Therefore, we hypothesized that the stable expression of EBV-miR-BART7-3p may also promote stem-like properties of NPC. We first detected the presence of side population (SP) cells, which were reported as primitive stem cell-like populations, in the control and miR-BART7-3p-overexpressing cells. SP cells are identified using dual wavelength flow cytometry combined with Hoechst 33342 dye efflux, owing to the expression of one or more members of the ABC transporter family. They are also known to be long-lived, self-renewing and highly proliferative. The results showed that stable expression of EBV-miR-BART7-3p increased the percentage of SP cells (**Figure 1A**). In contrast, SPs were significantly reduced in EBV-positive cell lines (HONE1-EBV and HK1-EBV) in the presence of EBV-miR-BART7-3p inhibitor (**Supplementary Figure 2**, **Figure 1B**).When cultured on uncoated plates in serum-free stem cell medium, cells with stable expression of EBV-miR-BART7-3p had a higher chance to develop into free-floating tumor spheres and grew into a bigger size than the control cells (**Figure 1C**). The Western blot results showed that the forced overexpression of EBV-miR-BART7-3p led to an increase in the protein levels of the stem cell markers including ABCG2, OCT4, NANOG and sox-2 (**Figure 1D**). The transcriptional levels of these stem cell markers were also upregulated in the EBV-miR-BART7-3p-overexpressing cells compared to the vector control cells (**Supplementary Figure 3**). These findings implied that EBV-miR-BART7-3p could prompt stem cell markers expression and stem-like properties in NPC cells.

**Figure 2:**
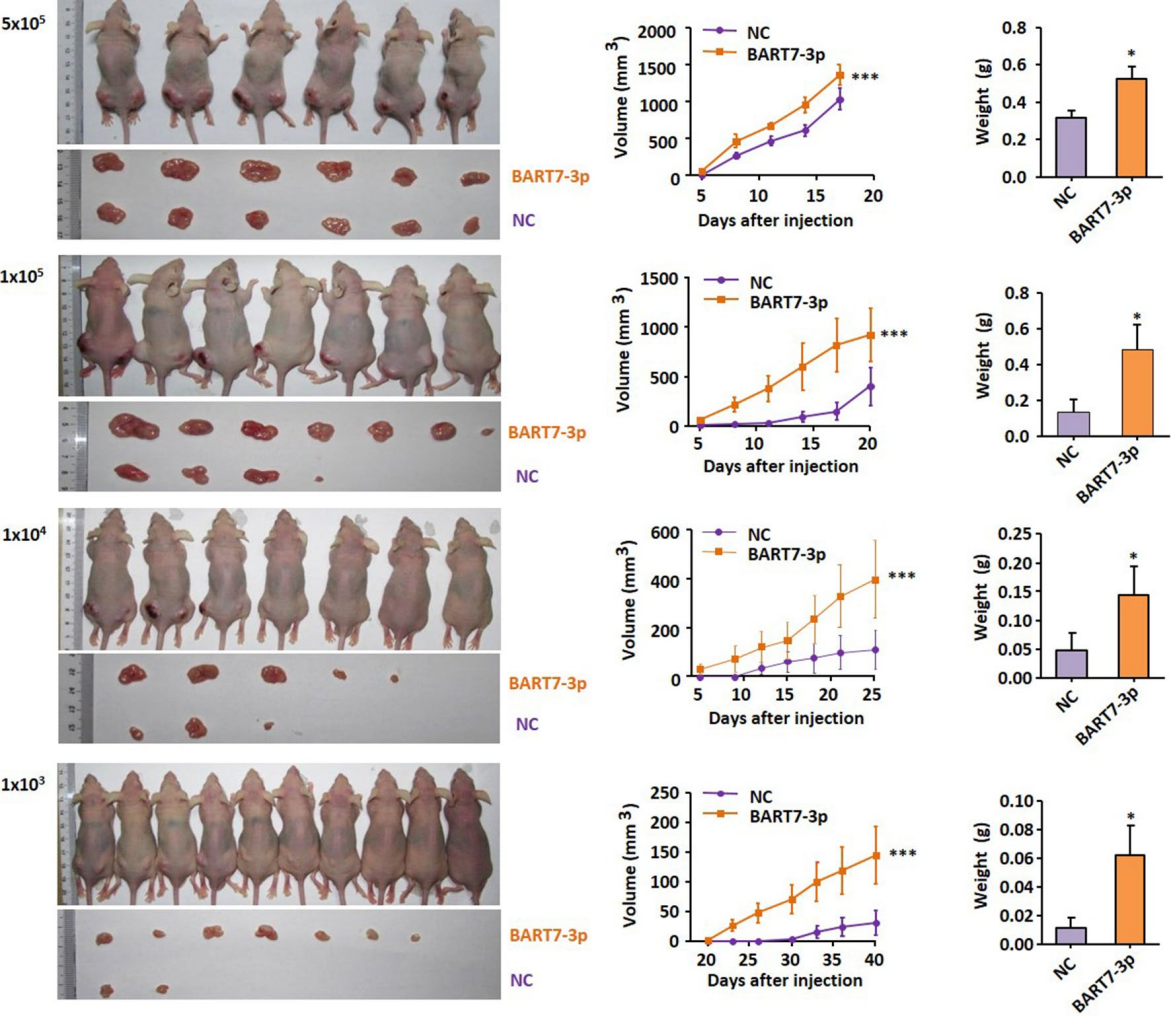
CNE2-NC and CNE2-BART7-3P were inoculated subcutaneously into the left and right sides of the back of each nude mouse respectively at concentration spanning a 4-point scale (1×10^3^, 1×10^4^, 1×10^5^, 5×10^5^). The volume or weigh of xenograft tumors were shown in the bar chart (*P < 0.05, ***P < 0.001).

To further assess the possible effects of EBV-miR-BART7-3p in enhancing stem-like or cancer-initiating properties of NPC cells, we have used *in vivo* limiting dilution assay to determine the cancer initiating cell frequency of CNE2-NC and CNE2-BART7-3P cells in immunocompromised murine xenograft models. A total of 1×10^3^, 1×10^4^, 1×10^5^ or 5×10^5^ CNE2-NC and CNE2-BART7-3P were injected subcutaneously into the left and right franks of each nude mouse respectively. Growth rates of the CNE2-BART7-3P tumors were significantly higher than those of controls injected with the same cell number. The tumors were also excised from the mice for photography and weighting. At each concentration, CNE2-BART7-3 always produced a statistically significant increase in the tumor initiating frequency compared to CNE2-NC (**Figure 2**), suggesting that stem cell proportion in BART7-3p cells might be higher. These observations suggest that EBV-miR-BART7-3p induced stem-like properties in NPC cells and enhanced the tumor-initiating cell population in CNE2 cells.

### EBV-miR-BART7-3p Inhibits SMAD7 Expression by Targeting Its 3'UTR

**Figure 3:**
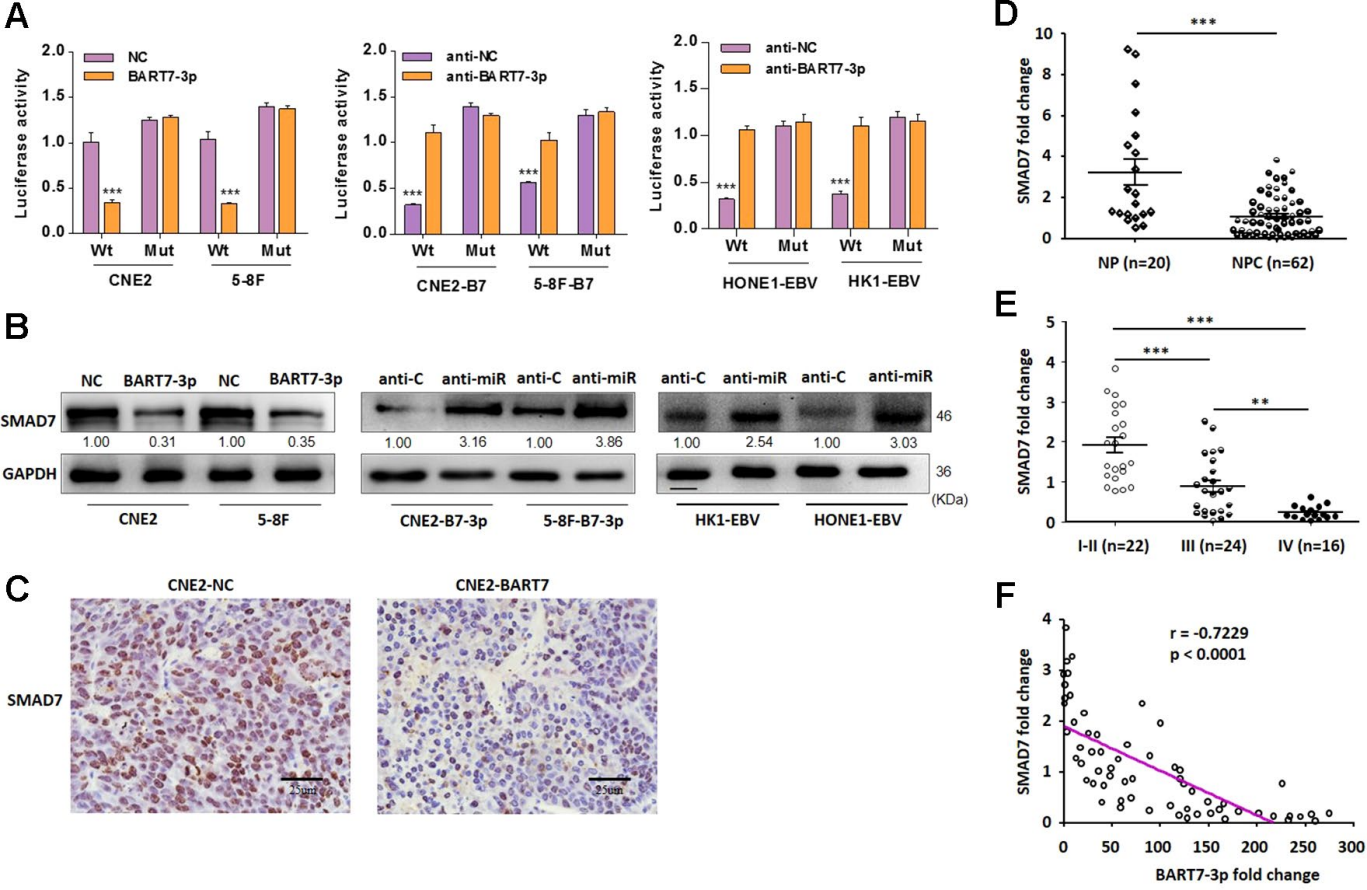
EBV-miR-BART7-3p induces stem-like properties in EBV-positive NPC cells. **(A)** CNE2 and 5-8F cells were cotransfected with EBV-miR-BART7-3p mimic or NC respectively, and luciferase reporters carrying either the predicted microRNA target site in SMAD7 3′UTR (wt) or its corresponding mutant (mut). Inhibition of EBV-miR-BART7-3p by anti-miR or anti-C was also performed. **(B)** Western blot analysis of endogenous SMAD7 protein expression levels in 5-8F and CNE2 cells treated with EBV-miR-BART7-3p or NC. Inhibition of EBV-miR-BART7-3p by anti-miR or anti-C was also performed. GAPDH served as the internal control. **(C)** SMAD7 expression was evaluated using immunohistochemistry assay in tumor tissues derived from mouse models and the control models (Magnification, ×400; scale bar, 25 um). **(D)** The levels of SMAD7 were detected by qPCR in NPC and NP tissue specimens. **(E)** SMAD7 mRNA expression was detected by qPCR in NPC samples (with clinical stage). **(F)** The levels of EBV-miR-BART7-3p and SMAD7 were detected by qPCR in NPC tissue specimens, normalized to the U6 snRNA and GAPDH, respectively. The correlation between EBV-miR-BART7-3p and SMAD7 expression levels was calculated (**P < 0.01, ***P < 0.001).

We have demonstrated that EBV-miR-BART7-3p could promote stem-like properties of NPC cells in both *in vitro* and mouse models. Next we explored the underlying mechanism by firstly performing bioinformatics search for the potential targets of EBV-miR-BART7-3p. RNAhybrid showed a complementary match in nucleobase sequence between EBV-miR-BART7-3p seed sequence and the 3′UTR of SMAD7 (**Supplementary Figure 4A**). To examine the targeting effect of EBV-miR-BART7-3p to SMAD7, we assessed the luciferase activities of NC and BART7-3p cells which were either co-transfected with SMAD7 3′UTR (wt 3′UTR) or the mutant sequence (mt 3′UTR). Interestingly, the luciferase activity of the wt SMAD7 3′UTR but not the mutant 3′UTR was significantly reduced by miR-BART7-3p mimic while the luciferase activity of the wt SMAD7 3′UTR could not be reduced by the control mimic (NC) (**Figure 3A**). Anti-miR increased the luciferase activity of reporter with wt SMAD7 3′UTR but not of mt 3′UTR (**Figure 3A**). Moreover, to further validate the interaction between BART7-3p and SMAD7, we designed three separate sets of comparative experiments (NC vs. BART-3p in CNE2 and 5-8F cell, anti-C vs. anti-miR in CNE2-b7-3p and 5-8F-b7-3p cells, and anti-C vs. anti-miR in HK1-EBV and HONE1-EBV cells). The effects of EBV-miR-BART7-3p on SMAD7 mRNA and protein expression were also examined in NPC cell lines. Upregulation of BART7-3p led to a decrease in the expression level of SMAD7 protein in CNE2-BART7-3p, 5-8F-BART7-3p, HK1-EBV and HONE1-EBV cell lines compared to the control as shown by qPCR and Western blot (**Figure 3B**, **Supplementary Figure 4B**). Importantly, the expression of SMAD7 was obviously reduced in the mouse tumors derived from CNE2-BART7-3p cells comparing to the CNE2-NC cells (**Figure 3C**).

Further investigation on the relationship between EBV-miR-BART7-3p and SMAD7 was performed in 62 NPC and 20 NP tissue samples. Firstly, we noticed a significant decrease in the expression of SMAD7 at mRNA level in the NPC samples than in NP samples as revealed by qPCR (**Figure 3D**). Secondly, detection of the SMAD7 mRNA showed that the expression of SMAD7 was significantly lower in advanced clinical stages III–IV than in either early clinical stages I–II or NP (**Figure 3E**). Furthermore, a negative correlation in the expression levels between EBV-miR-BART7-3p and SMAD7 in 62 NPC tissue samples was observed (**Figure 3F**). Taken together, SMAD7 was shown to be a target gene of EBV-miR-BART7-3p, and the expression of SMAD7 in NPC clinical tissue was inversely correlated with the expression of BART7-3p.

### EBV-miR-BART7-3p Upregulates the Expression of TGF-β Receptor and Induces the Nuclear Translocalization of P-SMAD2 and P-SMAD3 by Suppressing SMAD7

**Figure 4:**
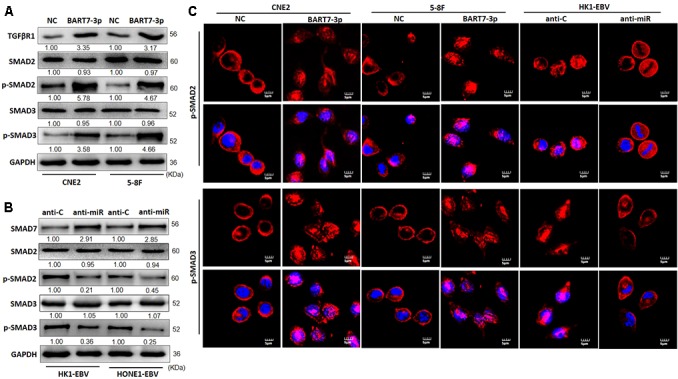
EBV-miR-BART7-3p stimulates TGF-β signaling and induces the relocalization of p-SMAD2 and p-SMAD3 by suppressing SMAD7. **(A)** The expression of TGF-β-R1, SMAD2, p-SMAD2, SMAD3, and p-SMAD3 was detected by Western blot in indicated cells treated with lentiviral particles carrying EBV-miR-BART7-3p and their corresponding NCs, respectively. GAPDH served as the internal control. **(B)** Western blot analysis of endogenous SMAD7, p-SMAD2, p-SMAD3 protein expression levels in HK1-EBV and HONE1-EBV cells treated with anti-miR or anti-C. GAPDH served as the internal control. **(C)** Confocal analysis was used to compare the expression and nuclear accumulation of p-SMAD2 and p-SMAD3 between NC cells and BART7-3p NPC cells (Magnification, ×1200; scale bar, 5 um).

It is well known that TGF-β signaling can be modulated by SMAD7 in performing stem-like properties and EMT (Ten and Hill, 2004; Derynck et al., 2014). Hence, we next elucidated whether EBV-miR-BART7-3p could regulate TGF-β signaling pathway by suppressing SMAD7. Western blot showed that the upregulation of BART7-3p led to an increase in the expression level of TGF-β-R1, p-SMAD2 and p-SMAD3 while the expression of SMAD2 and SMAD3 had no apparent change in either CNE2-BART7-3p or 5-8F-BART7-3p cells compared to their control counterparts (**Figure 4A**). Knockdown of BART7-3p in EBV-infected cell lines (including HK1-EBV and HONE1-EBV) led to an increase in SMAD7 at protein level, while a reduction in the expression of p-SMAD2 and p-SMAD3 (**Figure 4B**). Together, EBV-miR-BART7-3p activates TGF-β signaling by targeting expression of SMAD7.

It has been reported that p-SMAD2 and p-SMAD3 could directly combine with co-SMADs in cell nucleus and influence genes expression. To investigate whether BART7-3p might affect the localization of p-SMAD2 and p-SMAD3 in NPC cells, we detected the localization of these SMAD proteins using confocal microscopy. We observed that p-SMAD2 and p-SMAD3 were highly expressed and accumulated in the nuclei of NPC cells upon exogenic expression of EBV-miR-BART7-3p (**Figure 4C**). Collectively, EBV-miR-BART7-3p might promote the stem-like phenotypes in NPC cells by suppressing SMAD7 and stimulating TGF-β signaling.

### Knockdown of SMAD7 Resembles the Phenotypes Generated by the EBV-miR-BART7-3p

**Figure 5:**
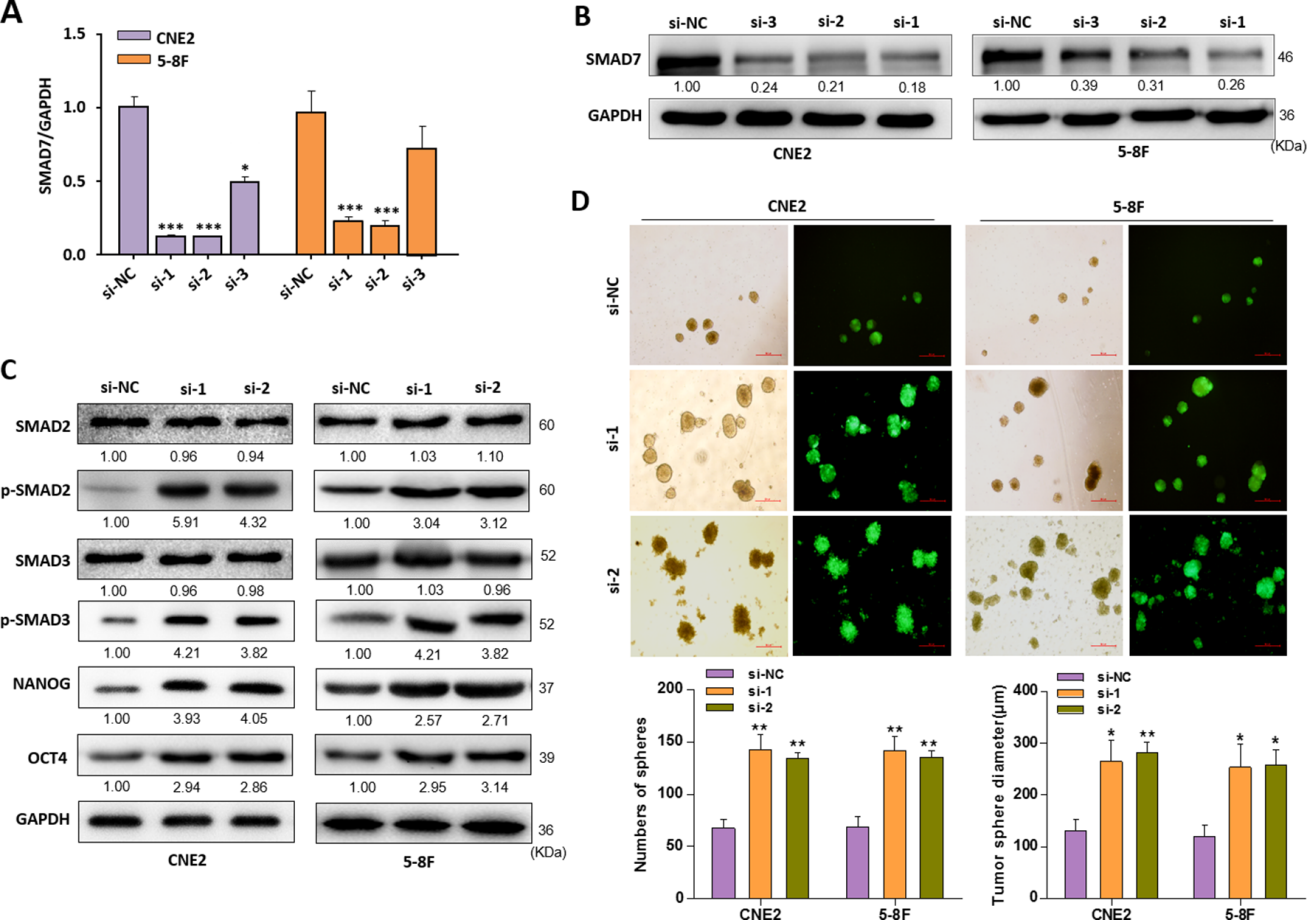
Knockdown of SMAD7 mimicked the EBV-miR-BART7-3p-induced phenotype. **(A**, **B)** CNE2 and 5-8F cells treated with siRNAs (si-1, si-2, si-3) targeting SMAD7 were analyzed by Western blot and qPCR. GAPDH served as a loading control. **(C)** The expression of p-SMAD2, p-SMAD3 and stem-cell markers (NANOG, OCT4) were determined by Western blot in CNE2 and 5-8F cells treated with si-1 and si-2 against SMAD7 or si-NC. GAPDH was internal control. **(D)** Images showing tumor sphere formation in either si-SMAD7 or si-NC NPC cells (Magnification, ×20; scale bar, 500 um). Columns in chart show number of spheres counted in 9 fields for each group. (*P < 0.05, **P < 0.01, ***P < 0.001).

Further experiments were performed to elucidate that SMAD7 is the mediator in activating TGF-β signaling and inducing stem-like phenotypes in NPC cells. siRNAs (siRNA-1, siRNA-2, siRNA-3) were used to silence SMAD7 expression in CNE2 and 5-8F cells (**Figures 5A, B**). siRNA-1 and siRNA-2 were chosen to be used in the following experiments as it could potently suppress the SMAD7 at both mRNA and protein level. As expected, knockdown of SMAD7 could increase the expression of p-SMAD2 or p-SMAD3, as well as stem-cell markers such as NANOG and oct-4 (**Figure 5C**). The incidence rate in forming tumor spheres is higher in SMAD7-silenced NPC cells than control cells. The tumor spheres consisted of SMAD7-silenced NPC cells were also larger in volume compared to control cells (**Figure 5D**). These data suggested that knockdown of SMAD7 was able to induce a stem-like phenotype in NPC cells, which was similar with the phenotype induced by EBV-miR-BART7-3p overexpression.

### Functional Role of SMAD7 on the EBV-miR-BART7-3p Which Mediates Stemness Phenotypes in NPC Cells

**Figure 6:**
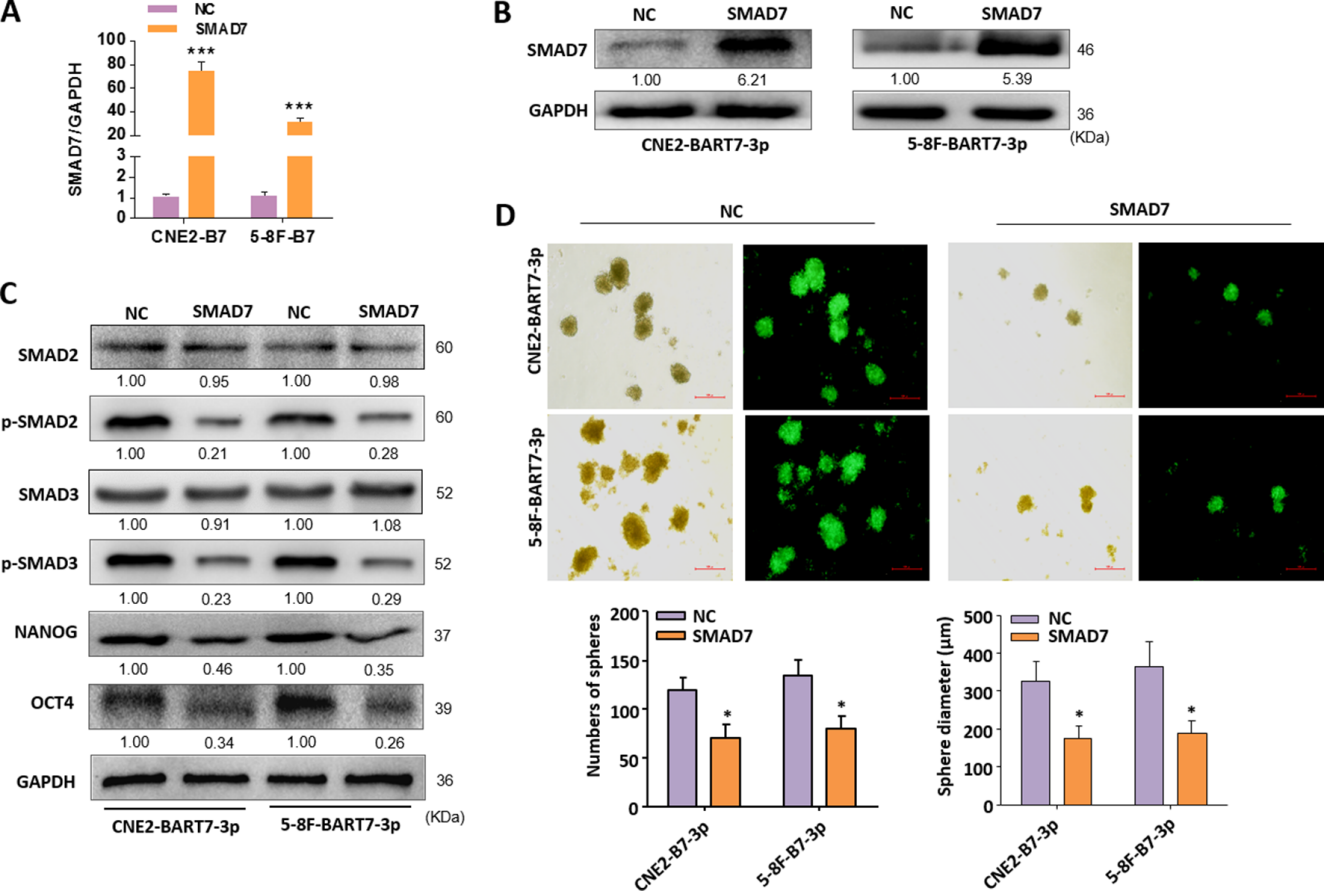
Functional validation of SMAD7 effects on EBV-miR-BART7-3p-mediated stemness phenotypes in NPC cells. **(A**, **B)** The expression of SMAD7 was detected by qPCR and Western blot in indicated cells transfected by SMAD7 expression plasmids. **(C)** The expression of p-SMAD2, p-SMAD3, NANOG and OCT4 in CNE2-BART7-3p and 5-8F-BART7-3p cells was measured by Western blot in the presence and absence of SMAD7. GAPDH served as the internal control. **(D)** Images showing tumor sphere formation in either CNE2-BART7-3p or 5-8F-BART7-3p cells transfected by SMAD7 expression plasmids (Magnification, ×20; scale bar, 500 um). Columns in chart show number of spheres counted in 9 fields for each group. (*p < 0.05, ***p < 0.001).

The above data demonstrated that BART7-3p cells could inhibit SMAD7 expression, and thus inducing the stem-like characteristic in NPC cells. The next step was to restore the expression of SMAD7 in BART7-3p cells by transfecting them with an expression plasmid of SMAD7. Successful rescue of SMAD7 in CNE2-BART7-3p and 5-8F-BART7-3p cells was observed (**Figures 6A, B**) and it down-regulated p-SMAD2, p-SMAD3, NANOG and oct-4 at protein level (**Figure 6C**). Tumor spheres analyses were used to further examine the stemness phenotypes of NPC cells which were transfected with SMAD7 expression plasmids. The data showed that CNE2-BART7-3p or 5-8F-BART7-3p cells which were transfected with SMAD7 plasmids had lower efficiency in forming tumor spheres than their counterparts which were transfected with control vectors. The spheres formed by SMAD7-reconsititued cells were also smaller in size (**Figure 6D**). Together, we demonstrated that down-regulation of SMAD7 as a targeting result of EBV-miR-BART7-3p induced stem cell phenotype in NPC cell lines.

### EBV-miR-BART7-3p Reduces the Sensitivity of NPC Cells to Chemotherapeutic Drugs

**Figure 7:**
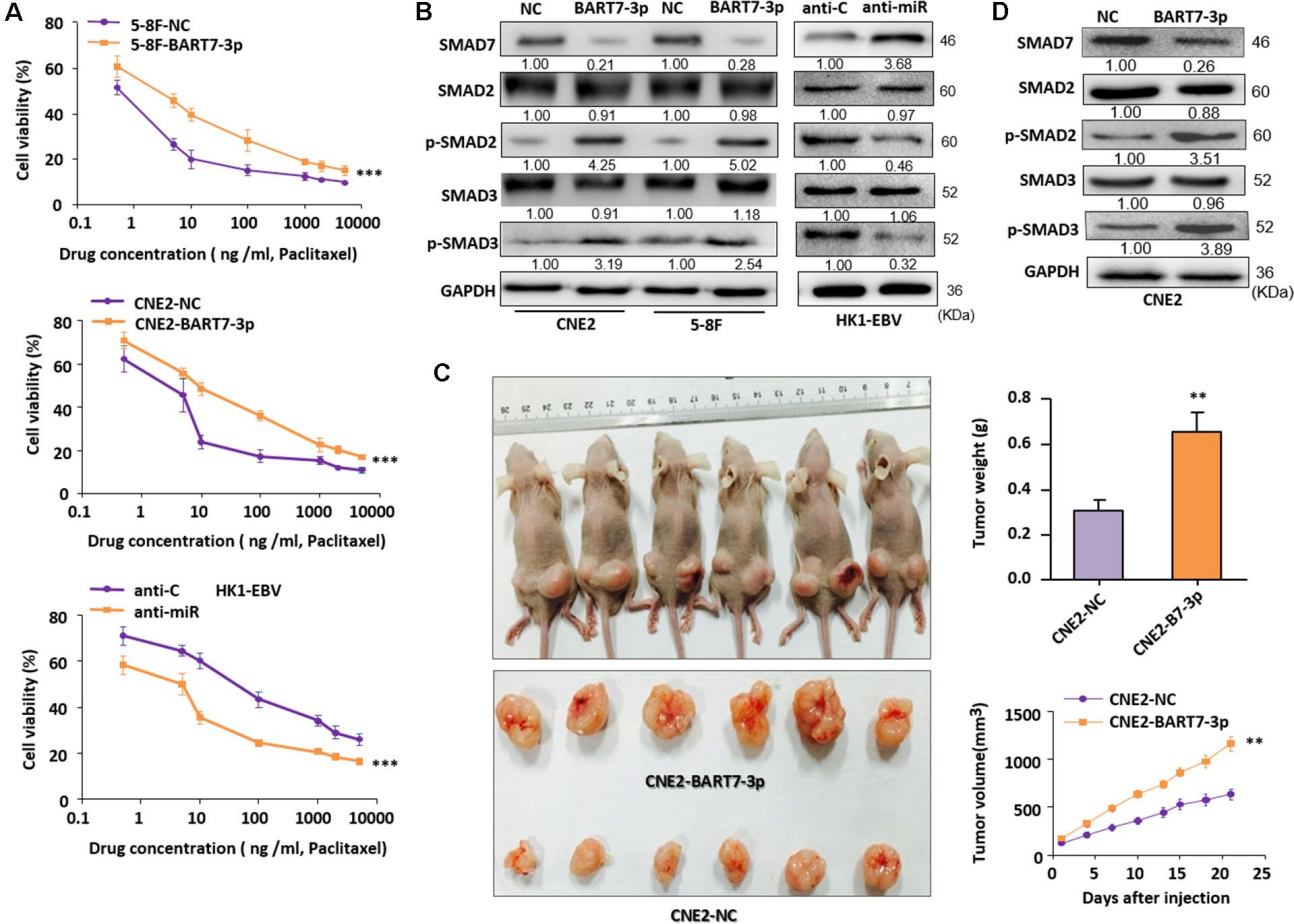
EBV-miR-BART7-3p reduces the sensitivity of chemotherapeutic drugs of NPC cells. **(A)** Cell viabilities of either CNE2-BART7-3p or 5-8F-BART7-3p cells treated by three chemotherapy drugs (fluorouracil, cisplatin, paclitaxel) with varied density were determined by OD values. HK1-EBV cells transfected with anti-miR or anti-C cells were also measured. **(B)** Western blot analyses of SMAD7 and p-SMAD2/3 in indicated cells treated with cisplatin (5 mg/kg). **(C)** As the pictures shown, xenograft tumors contained CNE2-BART7-3p grew faster and bigger even under the condition of cisplatin (5 mg/kg) applied every 3 days after tumors grew to 10 mm, compared to NC. The volume or weigh of xenograft tumors were shown in the bar chart. **(D)** Western blot analyses of SMAD7 and p-SMAD2/3 in indicated cells extracted from xenograft tumors in nude mice. (**p < 0.01, ***p < 0.001)

Cancer stem cells have been reported to have higher drug resistance and therefore can escape from tumor therapy and cause cancer relapse. We strived to know if EBV-miR-BART7-3p would upregulate the resistance of NPC cells to the chemotherapy that were widely used to treat the patients. MTT assay showed that the sensitivity of BART7-3p cells was lower than control cells in response of paclitaxel, fluorouracil and cisplatin treatments. In contrast, a decrease in the cell viability percentage of HK1-EBV cell line in the drug treatment was noticed when EBV-miR-BART7-3p was inhibited (**Figure 7A**, **Supplementary Figure 5**). p-SMAD2 and p-SMAD3 levels were increased in CNE2-BART7-3p, 5-8F-BART7-3p and HK1-EBV cells treated with paclitaxel by Western blot (**Figure 7B**). Next, we subcutaneously injected 10^6^ CNE2-BART7-3p or 10^6^ NC cells into nude mice. After the tumors grew to 10 mm in diameter, intraperitoneal injection of cisplatin (5 mg/kg) was applied every 3 days. As the picture shown, tumors formed by BART7-3p cells grew faster and larger in compared to the controls (**Figure 7C**). The results showed that SMAD7 protein level was much lower in the extracted tumors formed by BART7-3p cells than NC cells. However, p-SMAD2 and p-SMAD3 levels were much higher in the extracted xenograft tumors formed by BART7-3p cells (**Figure 7D**). All these suggest that EBV-miR-BART7-3p could increase the chemotherapy (cisplatin) resistance to NPC cells *in vivo* by targeting SMAD7.

## Discussion

NPC is frequently presented with cervical lymph node metastasis in early stage and local recurrence or distant metastasis in advanced stage. Therefore, it is important to study the molecular mechanism of metastasis, recurrence and chemo-resistance, which are the major causes of treatment failure and poor prognosis of NPC. Cancer stem-like cells are thought to contribute to cancer recurrence, metastasis and resistance of chemoradiotherapy (Wang et al., 2013; Easwaran et al., 2014). *EMT* is an example of cellular plasticity, driving invasion, therapeutic resistance, and *stemness* of cancer cells. Altered epithelial functions enable the cancer stem-like cells to survive and exhibit resistance to growth inhibitory drugs, thereby contributing to long-term cancer recurrence and progression. In a previously published paper, we have confirmed that upregulated EBV-miR-BART7-3p induced the EMT property in NPC. Here, we further showed that EBV-miR-BART7-3p was able to enhance NPC stemness and led to NPC recurrence or treatment failure.

In this study, we demonstrated that EBV-miR-BART7-3p enhanced the NPC cells to form more free-floating tumor spheres with increased size. The side population was also enriched in NPC cells with overexpression of EBV-miR-BART7-3p. In addition, a higher *in vivo* tumor formation rate and expression of stem cell markers were observed in EBV-miR-BART7-3p-expressing cells than the control cells. These findings for the first time suggest that the stem-like signatures in NPC were promoted by EBV-miR-BART7-3p. A further validation supported that silencing of endogenous EBV-miR-BART7-3p in EBV-positive NPC cell line led to a decrease in the stem cell phenotypes. Furthermore, EBV-miR-BART7-3p-expressing cells displayed a greater drug resistance as demonstrated by their higher levels of cell viabilities than those of the control cells after treated with fluorouracil, cisplatin, paclitaxel. This study suggests a potential application of EBV-miR-BART7-3p as a novel therapeutic marker for clinical management, especially for detecting the patients with high risks of metastasis or recurrence.

SMAD7, a member of I-SMADs, participates in TGF-β signaling at the transcriptional level (Itoh and Ten, 2007; Zunwen et al., 2012). It was known to modulate differentiation of myocyte and adipocyte and induce metastasis of tumor cells. It is also reported that SMAD7 induces mouse embryonic stem cell pluripotency by regulating STAT3 signaling (Yu et al., 2017). Recent studies have showed that several cellular microRNAs (such as miR-21, miR-182, miR-106b) could regulate the expression of SMAD7 in various cancers. MicroRNA-21 promotes the bone formation of bone marrow mesenchymal stem cells (BMMSCs) while this process is regulated, in part, by the SMAD7-SMAD1/5/8-Runx2 pathway (Li et al., 2017a). MicroRNA-182 targets SMAD7 to potentiate TGF-β-induced EMT and metastasis of cancer cells (Yu et al., 2016). MicroRNA-106b was also known to regulate EMT by the TGF-β/SMAD signaling pathway, implicating a role in the development of cancer stem-like cells (Cheng et al., 2016). However, viral microRNAs have not been reported to directly inhibit SMAD7. This study has proved that a viral microRNA directly suppressed the expression of SMAD7 by binding to its 3′UTR. Re-expression of SMAD7 could reverse EBV-miR-BART7-3p-induced phenotypes.

SMAD7 may not be the only target regulated by EBV-miR-BART7-3p in promoting stem. It is well known that microRNA usually governs a set of targets simultaneously. Our previous study found that EBV-miR-BART7-3p activated the PI3K/AKT signaling pathway to promote metastasis and EMT of NPC cells by targeting PTEN (Cai et al., 2015a), suggesting PTEN may also induce cancer stem cell formation in NPC as reported by other articles (Matsuda et al., 2018), so SMAD7 may not be the unique target of EBV-miR-BART7-3p in NPC. However, it is still unclear which associated pathway is the main factor inducing cancer stem cell formation in EBV-infected NPC and whether these two target genes play roles independently or synergistically. It is interesting to further explore this in the future.

TGF-β signaling pathway plays a very complex role in carcinogenesis with a biphasic action by initially suppressing tumorigenesis but promoting tumor progression in the later stages. LMP1, LMP2A and miR-BART7-3p have been reported to suppress TGF-β signaling (Prokova et al., 2002; Fukuda and Longnecker, 2004; Gao et al., 2017). However, some recent reports showed that TGFR-1 was up-regulated in primary NPC tissues and positively correlated with disease staging (Xu et al., 1999; Hu et al., 2012; Ma et al., 2017). EBNA1 and LMP1 have been shown to stimulate the production of TGF-β, and yet, they disrupted the signal transduction rendering the cells refractory to the TGF-β-mediated cytostasis. Therefore, further understanding of some underlying mechanisms may provide a good explanation for these seemingly contradicting roles of EBV-encoded latent proteins in regulating the TGF-β pathway, knowing how EBV fine-tunes the response to TGF-β and achieves malignant transformation in different phases or conditions.

Interestingly, it has been shown that TGF-β-induced EMT can drive tumor cells towards a more stem cell-like phenotype (Shipitsin et al., 2007; Anido et al., 2010). A decrease in stem cell number was also observed after treatment with TGF-βR1 inhibitors (Kondo et al., 2011). Therefore, it is now clear that high TGF-β production can promote aggressive phenotypes, but the contribution of EBV to this process is still not well-explored. This would be an important research area to be explored in EBV-associated cancers, particularly as EBV latent proteins (LMP1, LMP2A) have been shown to contribute to the induction and maintenance of cancer stem-like cell population in NPC (Kong et al., 2010). In this study, we discovered a viral microRNA related to NPC stemness, providing a new insight into EBV-miR-BART7-3p mediated regulatory mechanism of TGF-β signaling in NPC *via* targeting SMAD7 (**Supplementary Figure 6**). Nuclear expression and translocation of p-SMAD2 and p-SMAD3 were up-regulated in EBV-miR-BART7-3p-expressing cells (**Supplementary Figure 7**).

This study discovers a novel viral miRNA-mediated mechanism underlying the cancer stem-like properties. Activin receptor and/or nodal is one of important pathways through SAMD proteins (Schier, 2009). They often act as morphogens and is regulated by multiple mechanisms, including extracellular antagonists (Lefty1/2, Cerberus, Follistatin), agonists (CRIPTO1), processing enzymes (Spc1, Spc4), and intracellular molecules including SMAD6/7. Although several reports suggested that activin and/or nodal play roles in regulating stem cell pluripotency (Oshimori and Fuchs, 2012), the function of activin/nodal is less clear (Cambray et al., 2012; Wu et al., 2016). In this study, we did not fully demonstrate this in EBV-associated cancers, so further research will be necessary to demonstrate the importance of the associated underlying mechanism in the self-renewal or differentiation and in the formation of CSCs.

Taken together, EBV-miR-BART7-3p plays vital roles in the NPC stemness by directly suppressing SMAD7, which leads to an activated TGF-β signaling. Most importantly, this study suggests a clinical significance that EBV-miR-BART7-3p may be a potential target of chemoradiotherapy resistance in NPC clinical treatment.

## Data Availability Statement

The raw data supporting the conclusions of this manuscript will be made available by the authors, without undue reservation, to any qualified researcher.

## Ethics Statement

Patient’s informed written consents and the Ethics Committee approval of Southern Medical University have been received before collecting clinical tissues. The Ethical Committee for Animal Research of the Southern Medical University approved our animal experiments (No.44007200042655), which were organized by the state guidelines from the Ministry of Science and Technology of China.

## Author Contributions

LC, XL, CT, and WCC: Study design and literature guide. LC, XL, YFL, and TC: Development of methodology. LC, YFL, and TC: Acquisition of data. XZ, YL, YC, XL, WCC, JW, and TD: Analysis and interpretation of data. WCC, LC, YFL, TC, CT, WC, WZ, and HZ: Writing, review and revision of the manuscript. XL, CT, and XML: Administrative, technical or material support. XL: Study supervision. All authors read and approved the final manuscript.

## Funding

This study was financially supported by grants from the National Natural Science Foundation of China (81602530, 81572644, 81773111), the National Natural Science Foundation of China/Research Grants Council Joint Research Scheme (81861168033), the Natural Science Foundation of Guangdong Province (2016A030310272), the Shenzhen Key Laboratory of Viral Oncology (ZDSYS201707311140430), and the Shenzhen Science and Technology Key Project (JCYJ20170413165531148). Finally, we are very grateful to the Sanming Project of Medicine in Shenzhen (No. SZSM201612023) for the contribution to this research.

## Conflict of Interest

The submitted work was not carried out in the presence of any personal, professional or financial relationships that could potentially be construed as a conflict of interest.

## Supplementary Material

Supplementary Figure 1 **(A)** 5-8F and CNE2 cells transfected with EBV-miR-BART7-3p\lentivirus vectors were observed under visible light and fluorescence microscope (200×). Cells treated by EBV-miR-ctrl/lentivirus vectors were observed under visible light and fluorescence microscope (200×). **(B)** The expression levels of EBV-miR-BART7-3p in CNE2, 5-8F and NPC tissue derived from clinical patients (***P < 0.001).

Supplementary Figure 2 The expression levels of stem cell markers (ABCG2, NANOG, OCT 4, SOX2) in the indicated CNE2 and 5-8F cells (*P < 0.05, **P < 0.01, ***P < 0.001).

Supplementary Figure 3 EBV-miR-BART7-3p/U6 expression in EBV-positive cell lines (HONE1-EBV and HK1-EBV) in the presence of EBV-miR-BART7-3p inhibitor (***P < 0.001).

Supplementary Figure 4 **(A)** The nucleobase sequence between EBV-miR-BART7-3p seed sequence and the 3′UTR of SMAD7 showed by RNAhybrid. **(B)** Smad7 expression in both CNE2-BART7-3p and 5-8F-BART7-3p cell lines compared to the control showed by qPCR (*P < 0.05, **P < 0.01).

Supplementary Figure 5 The cell viability of CNE2, 5-8F and HK1-EBV cell lines in the drug treatment (fluorouracil, cisplatin) presented by MTT assay (***P < 0.001).

Supplementary Figure 6 The expression levels of several downstream genes (AP-1/FOXH1/RUNX2/CBFA3) of TGF-β in the indicated CNE2 and 5-8F cells by qPCR assay (***P < 0.001).

Supplementary Figure 7 The expression levels of SMAD7 and p-STAT3 in the indicated CNE2 and 5-8F cells by Western blot.

Supplementary Table 1 The demographic information of clinical samples.

Supplementary Table 2 Clinicopathological characteristics of nasopharyngeal carcinoma patients.

Supplementary Table 3 Sequences of primers for lentivirus vector and BART7-3p mimic/inhibitor.

Supplementary Table 4 Sequences of primers for qPCR.

Supplementary Table 5 The information of antibodies.

Supplementary Table 6 Sequences of primers for SMAD7 siRNA.
