# Supplementary material for: EBV-miR-BART7-3p Imposes Stemness in Nasopharyngeal Carcinoma Cells by Suppressing SMAD7
Source: Front Genet. 2019 Oct 17;10:939. doi: 10.3389/fgene.2019.00939 (PMC6811651; doi:10.3389/fgene.2019.00939)
Supplement: Supplementary Figure 1 — (A) 5-8F and CNE2 cells transfected with EBV-miR-BART7-3p\lentivirus vectors were observed under visible light and fluorescence microscope (200×). Cells treated by EBV-miR-ctrl/lentivirus vectors were observed under visible light and fluorescence microscope (200×). (B) The expression levels of EBV-miR-BART7-3p in CNE2, 5-8F and NPC tissue derived from clinical patients (***P < 0.001). [file Presentation_1.ppt]

## Slide 1
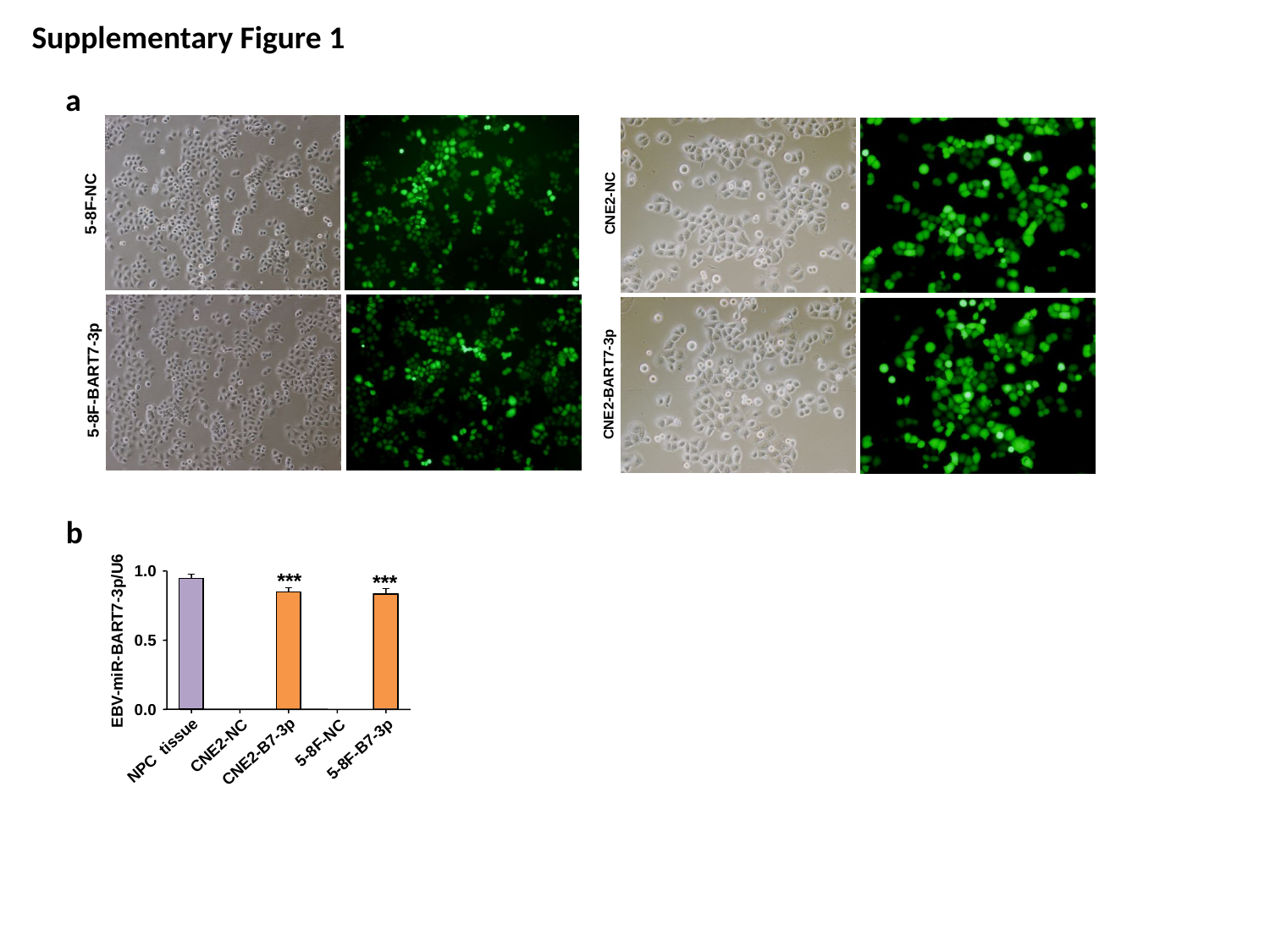

Supplementary Figure 1
a
5-8F-NC
CNE2-NC
5-8F-BART7-3p
CNE2-BART7-3p
b
1.0
***
***
0.5
EBV-miR-BART7-3p/U6
0.0
5-8F-NC
CNE2-NC
5-8F-B7-3p
CNE2-B7-3p
 NPC tissue

## Slide 2
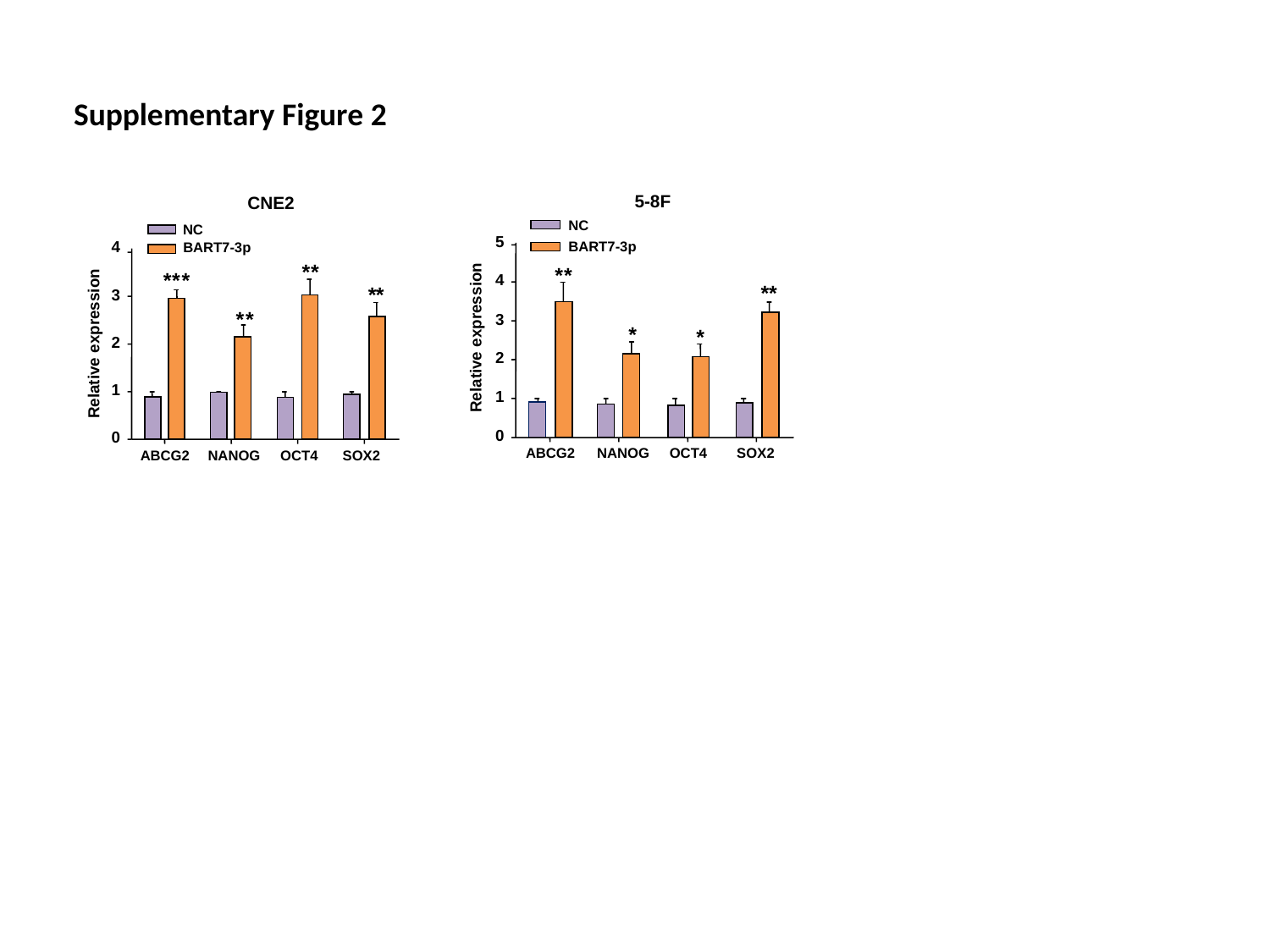

Supplementary Figure 2
5-8F
NC
5
BART7-3p
*
*
4
*
*
3
*
*
Relative expression
2
1
0
ABCG2
NANOG
OCT4
SOX2
CNE2
NC
4
BART7-3p
*
*
*
*
*
*
*
3
*
*
2
Relative expression
1
0
ABCG2
NANOG
OCT4
SOX2

## Slide 3
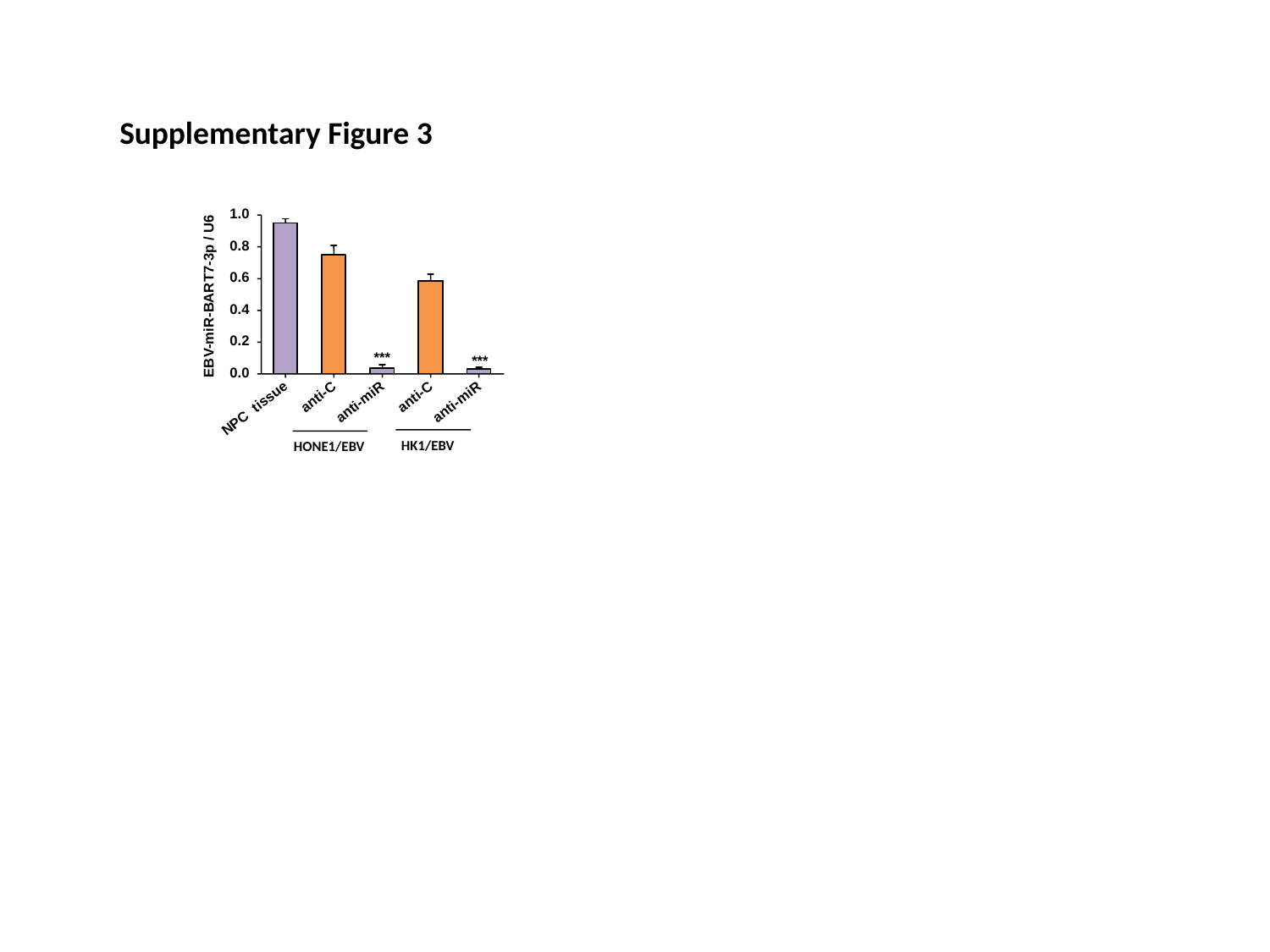

Supplementary Figure 3
1.0
0.8
0.6
EBV-miR-BART7-3p / U6
0.4
0.2
***
***
0.0
anti-C
anti-C
anti-miR
anti-miR
 NPC tissue
HK1/EBV
HONE1/EBV

## Slide 4
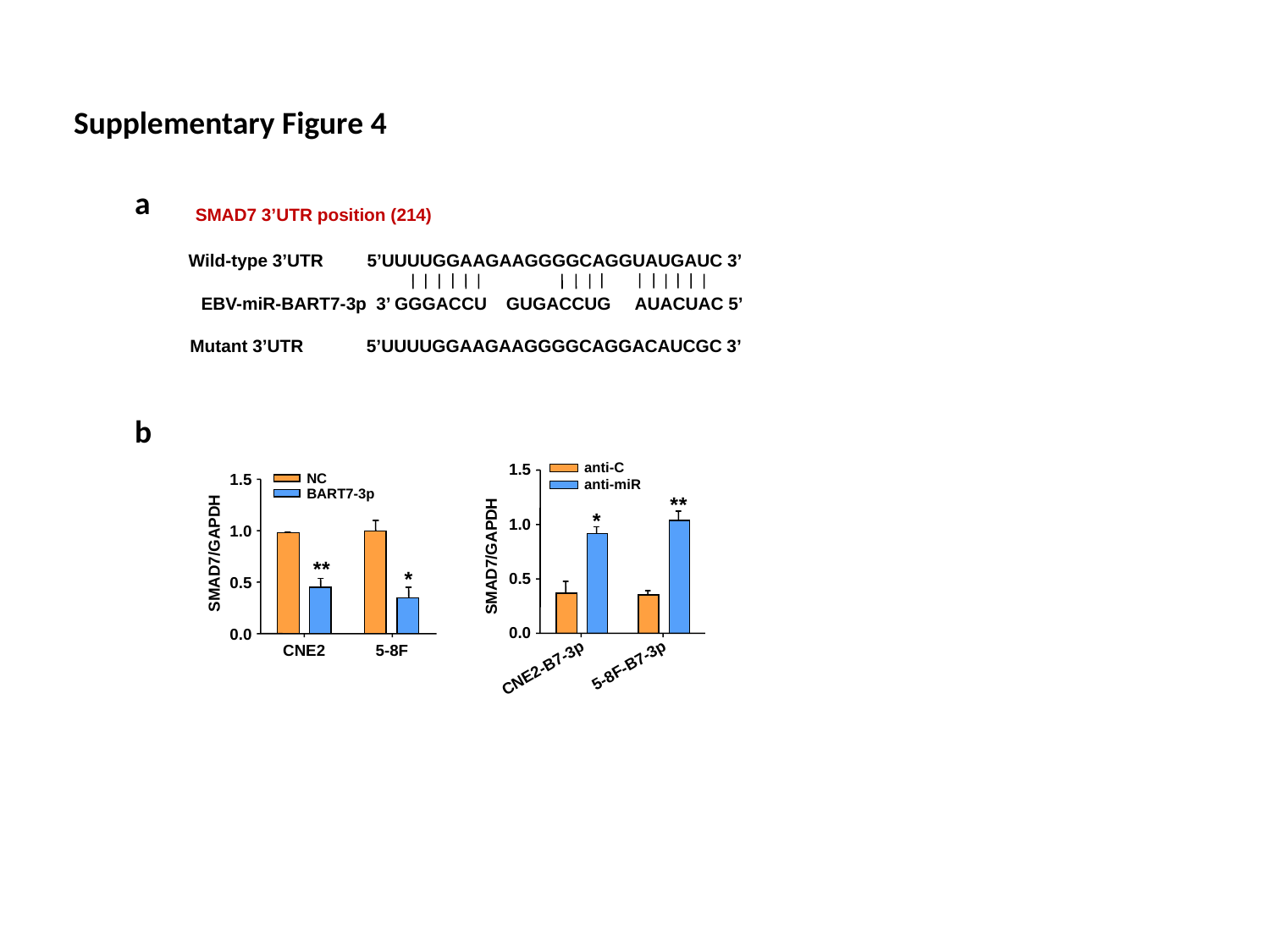

Supplementary Figure 4
a
SMAD7 3’UTR position (214)
Wild-type 3’UTR 5’UUUUGGAAGAAGGGGCAGGUAUGAUC 3’
EBV-miR-BART7-3p 3’ GGGACCU GUGACCUG AUACUAC 5’
Mutant 3’UTR 5’UUUUGGAAGAAGGGGCAGGACAUCGC 3’
b
anti-C
1.5
anti-miR
*
*
*
1.0
SMAD7/GAPDH
0.5
0.0
5-8F-B7-3p
CNE2-B7-3p
NC
1.5
BART7-3p
1.0
SMAD7/GAPDH
*
*
*
0.5
0.0
CNE2
5-8F

## Slide 5
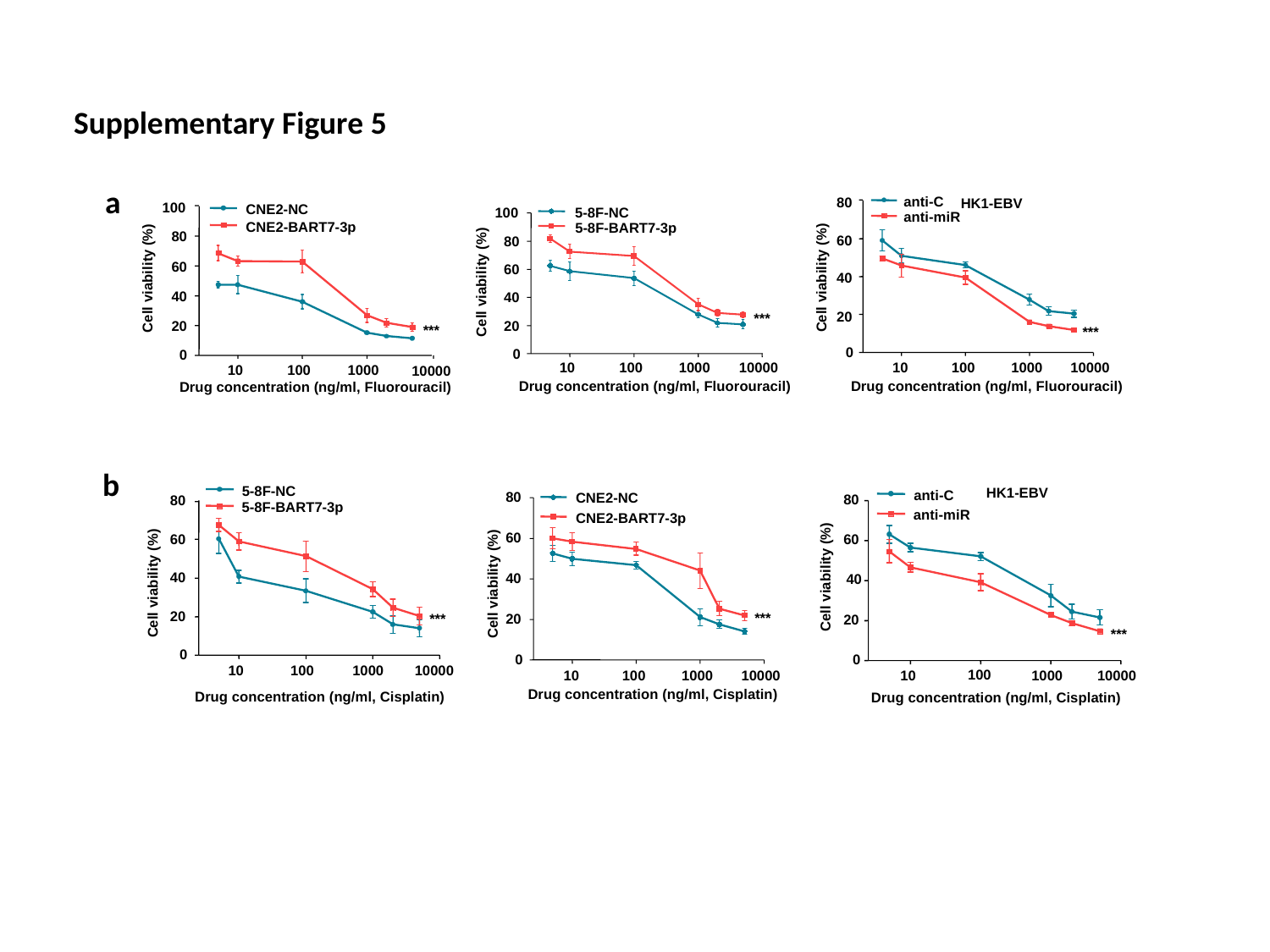

Supplementary Figure 5
a
anti-C
80
HK1-EBV
anti-miR
60
40
Cell viability (%)
20
***
0
10
100
1000
10000
Drug concentration (ng/ml, Fluorouracil)
100
CNE2-NC
CNE2-BART7-3p
80
60
Cell viability (%)
40
20
***
0
10
100
1000
10000
Drug concentration (ng/ml, Fluorouracil)
100
5-8F-NC
5-8F-BART7-3p
80
60
Cell viability (%)
40
***
20
0
10
100
1000
10000
Drug concentration (ng/ml, Fluorouracil)
b
5-8F-NC
80
5-8F-BART7-3p
60
40
Cell viability (%)
20
***
0
10
100
1000
10000
Drug concentration (ng/ml, Cisplatin)
HK1-EBV
anti-C
80
anti-miR
60
Cell viability (%)
40
20
***
0
100
10
1000
10000
Drug concentration (ng/ml, Cisplatin)
80
CNE2-NC
CNE2-BART7-3p
60
40
Cell viability (%)
***
20
0
10
100
1000
10000
Drug concentration (ng/ml, Cisplatin)

## Slide 6
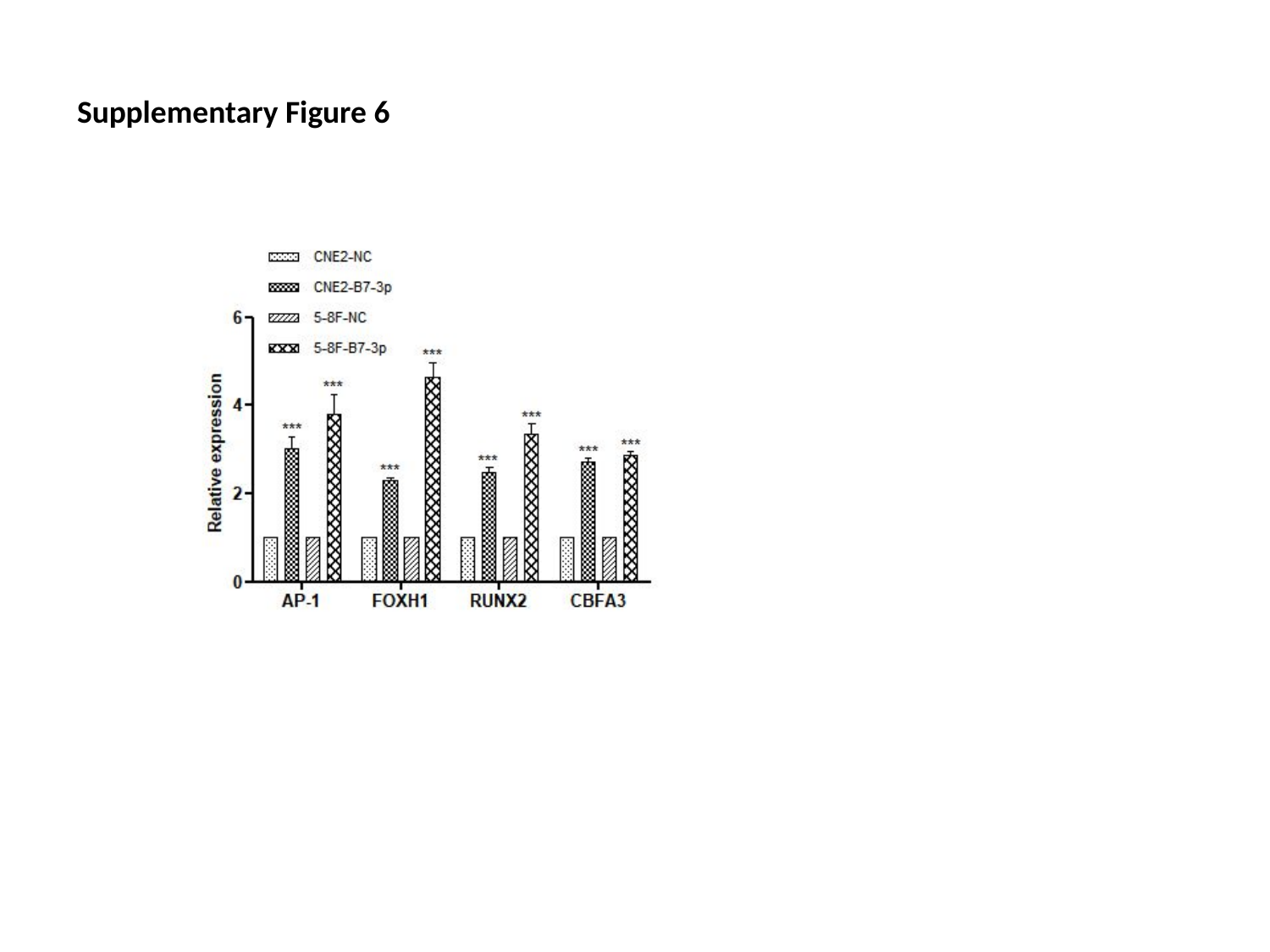

Supplementary Figure 6

## Slide 7
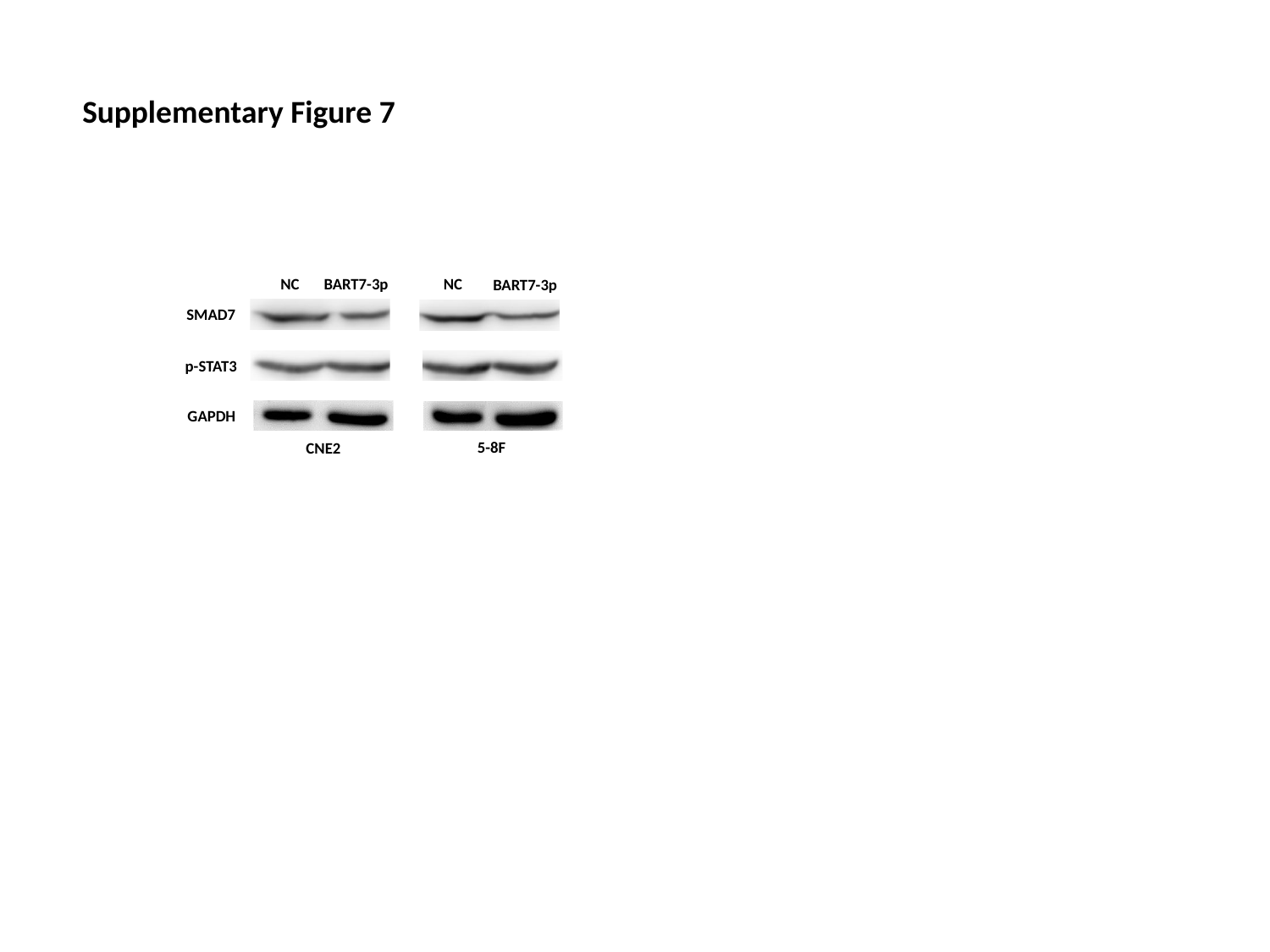

Supplementary Figure 7
BART7-3p
NC
NC
BART7-3p
SMAD7
p-STAT3
GAPDH
5-8F
CNE2
