# Supplementary material for: EBV-miR-BART7-3p Imposes Stemness in Nasopharyngeal Carcinoma Cells by Suppressing SMAD7
Source: Front Genet. 2019 Oct 17;10:939. doi: 10.3389/fgene.2019.00939 (PMC6811651; doi:10.3389/fgene.2019.00939)
Supplement: Supplementary Table 1 — The demographic information of clinical samples. [file DataSheet_1.doc]

**Supplementary Tables**

**Supplementary Table 1. The demographic information of clinical samples.**

| Characteristics | Clinical samples | | |
| --- | --- | --- | --- |
|  | NP (n=20) | NPC (n=62) | P value |
| Age, years | 47.4 | 52.33 | 0.1322^*^ |
| Gender, male | 12 | 42 | 0.5917^#^ |
| Poorly differentiated SCC |  | 2 |  |
| Undifferentiated cancer |  | 59 |  |
| Differentiated SCC |  | 1 |  |

^*^Independent t-test. ^#^Chi-square test. NP, Normal nasopharyngeal; NPC, Nasopharyngeal carcinoma; SCC, Squamous cell carcinoma

**Supplementary Table 2. Clinicopathological characteristics of nasopharyngeal carcinoma patients.**

| Characteristics | No. of patients (n=62) |
| --- | --- |
| Gender |  |
| Male | 42 |
| Female | 20 |
| Age (years) |  |
| ≤45 | 14 |
| >45 | 48 |
| Histological type |  |
| Poorly differentiated SCC | 2 |
| Undifferentiated cancer | 59 |
| Differentiated SCC | 1 |
| T stage |  |
| T1 | 22 |
| T2 | 11 |
| T3 | 16 |
| T4 | 13 |
| N stage |  |
| N0 | 12 |
| N1 | 17 |
| N2 | 18 |
| N3 | 15 |
| M stage |  |
| M0 | 58 |
| M1 | 4 |
| TNM stage |  |
| I | 3 |
| II | 19 |
| III | 24 |
| IV | 16 |

SCC, Squamous cell carcinoma

**Supplementary Table 3. Sequences of primers for lentivirus vector and BART7-3p mimic/inhibitor.**

| Vector/mimic/inhibitor | | Sense strand (5'-3') |
| --- | --- | --- |
| Lentivirus vector | Forward | GAGGATCCCCGGGTACCGGGTATTTTCCCATCAGCACCTG |
|  | Reverse | CACACATTCCACAGGCTAGTTCATTAGCTATCACAAAGCC |
| BART7-3p | mimic | CAUCAUAGUCCAGUGUCCAGGG |
|  | NC | UUCUCCGAACGUGUCACG |
|  | anti-miR | CCCUGGACACUGGACUAUGAUG |
|  | anti-C | CAGUACUUUUGUGUAGUACAA |

NC, negative control

**Supplementary Table 4. Sequences of primers for qPCR.**

| Gene | | Sense strand (5'-3') |
| --- | --- | --- |
| ABCG2 | Forward | CAGGTGGAGGCAAATCTTCGT |
|  | Reverse | ACCCTGTTAATCCGTTCGTTTT |
| NANOG | Forward | TTTGTGGGCCTGAAGAAAACT |
|  | Reverse | AGGGCTGTCCTGAATAAGCAG |
| OCT4 | Forward | CTGGGTTGATCCTCGGACCT |
|  | Reverse | CCATCGGAGTTGCTCTCCA |
| SOX2 | Forward | CTCGTGCAGTTCTACTCGTCG |
|  | Reverse | AGCTCTCGGTCAGGTCCTTT |
| SMAD7 | Forward | TTCCTCCGCTGAAACAGGG |
|  | Reverse | CCTCCCAGTATGCCACCAC |
| GAPDH | Forward | TGTGGGCATCAATGGATTTGG |
|  | Reverse | ACACCATGTATTCCGGGTCAAT |

**Supplementary Table 5. The information of antibodies.**

| Antibody | Cat. No | Company | Molecular weight | Dilution (WB/IHC) |
| --- | --- | --- | --- | --- |
| ABCG2 | SAB4300689 | Sigma | 65-80 kDa | 1:800/1:1000 |
| OCT4 | ab109183 | Abcam | 39 kDa | 1:500/1:1000 |
| NANOG | ab109250 | Abcam | 35 kDa | 1:1000 |
| SOX2 | ab192078 | Abcam | 43 kDa | 1:250 |
| SMAD7 | SAB4300689 | Sigma | 46 kDa | 1:500 |
| TGF-βR1 | ab31013 | Abcam | 56 kDa | 1:500 |
| SMAD2 | ab40855 | Abcam | 58 kDa | 1:1000 |
| p-SMAD2 | ab53100 | Abcam | 58 kDa | 1:300/1:1000 |
| SMAD3 | ab40854 | Abcam | 48 kDa | 1:800/1:1000 |
| p-SMAD2 | ab63403 | Abcam | 48 kDa | 1:500/1:1000 |
| β-actin | 5779-1 | Epitomics | 43 kDa | 1:1000 |
| GAPDH | 20301707-2 | Bioworld | 36 kDa | 1: 1000 |

**Supplementary Table 6. Sequences of primers for SMAD7 siRNA.**

| siRNA | | Sense strand (5'-3') |
| --- | --- | --- |
| siRNA-1 | Forward | CCAAUGACCACGAGUUUAUTT |
|  | Reverse | AUAAACUCGUGGUCAUUGGTT |
| siRNA-2 | Forward | AAAUCCAUCGGGUAUCUGGAG |
|  | Reverse | CCAGAUACCCGAUGGAUUUUC |
| siRNA-3 | Forward | UAGAUCAUAGAAGAUAUCCAG |
|  | Reverse | GGAUAUCUUCUAUGAUCUACC |
